# Postbiotics derived from recombinant lactic acid bacteria exhibit high IL6-binding capacity and suppress IL6-induced STAT3 signaling

**DOI:** 10.3389/fmicb.2025.1657810

**Published:** 2025-10-24

**Authors:** Abida Zahirović, Špela Zupančič, Andraž Verdir, Sebastjan Nemec, Slavko Kralj, Luka Snoj, Aleš Berlec

**Affiliations:** ^1^Department of Biotechnology, Jožef Stefan Institute, Ljubljana, Slovenia; ^2^Faculty of Pharmacy, University of Ljubljana, Ljubljana, Slovenia; ^3^Reactor Infrastructure Centre, Jožef Stefan Institute, Ljubljana, Slovenia; ^4^Department for Material Synthesis, Jožef Stefan Institute, Ljubljana, Slovenia; ^5^Reactor Physics Department, Jožef Stefan Institute, Ljubljana, Slovenia

**Keywords:** postbiotics, *Lactococcus lactis*, surface display, affibody, inflammatory bowel disease, cytokines

## Abstract

**Introduction:**

With growing evidence of clinical efficacy of probiotics in various diseases, safety concerns have arisen regarding the therapeutic use of live probiotic bacteria, especially in critically ill, immunocompromised, and pediatric populations. Serious probiotic-related adverse effects have been reported in these patients, including bloodstream infection and sepsis. This has led to an increased interest in developing postbiotics (non-viable bacterial products) that may exert beneficial effects on the host without the risks associated with administration of live microorganisms. The aim of this study was to explore postbiotic potential of recombinant *Lactococcus lactis* bacteria that have been engineered to display interleukin 6 (IL6)-targeting affibody (ZIL6) on their surface and are intended for treatment of inflammatory intestinal diseases.

**Methods:**

Five different killing treatments were applied to kill bacteria (heat, ethanol, sonication, UV, and gamma irradiation) and their effect on bacterial viability, morphology and functionality was examined *in vitro* using a combination of different techniques, including microscopy, flow cytometry, immunoassays and cell-based reporter assay.

**Results:**

The results showed that ZIL6 affibody displayed on *L. lactis* via non-covalent anchoring withstood the treatments applied to kill bacteria and remained functional after the loss of microbial viability. The degree of functionality was dependent on the type of treatment. Heat-killed cells retained 50% of the activity of live strain, while most of the activity was preserved after exposure of bacteria to ethanol, sonication, UV and gamma irradiation. The applied treatments varied in killing efficacy, whereby ethanol and heat rendered bacteria non-viable, UV and gamma irradiation yielded non-replicative cells, whereas sonication was ineffective in killing *L. lactis*. Among non-viable cells, ethanol-killed bacteria exhibited the greatest activity and showed high maximum binding capacity of 200 ng IL6 per mg dry cell weight, possessed strong nanomolar affinity for IL6, and inhibited up to 78% of IL6-induced STAT3 signaling.

**Conclusion:**

The study demonstrates that functional non-viable bacterial cells can be derived from the recombinant *L. lactis* with therapeutic proteins displayed on their surface and provides a good foundations for further studies of their postbiotic potential in adjunctive therapy of inflammatory intestinal diseases.

## 1 Introduction

During the last few decades, probiotics have been extensively investigated as an adjuvant therapy for intestinal diseases due to their ability to exert beneficial effects in the gastrointestinal tract (GIT) (van der Lelie et al., 2021). Mechanisms of action involve the modulation of the gut microbiota function, competitive exclusion of pathogens, regulation of immune responses, and reinforcement of the intestinal barrier (Mazziotta et al., 2023; Mazhar et al., 2024). Some probiotic strains can promote the repair of intestinal barrier damage caused by pathogens or proinflammatory cytokines (Chen et al., 2019). The clinical efficacy of probiotics has been demonstrated in diarrhea, pouchitis, ulcerative colitis, and necrotising enterocolitis (Wilkins and Sequoia, 2017). However, with a growing evidence supporting the use of probiotics in managing various diseases (Mazhar et al., 2025), safety concerns have been raised about the administration of live probiotic bacteria in high-risk patients, particularly in critically ill, immunocompromised, and pediatric populations (D'Agostin et al., 2021).

The main health risk of live probiotic bacteria is their potential to translocate from the intestine to systemic circulation and cause bloodstream infections with septic complications. While clinical trials have not reported instances of probiotic-related sepsis in healthy adults, there have been case reports of serious systemic infections caused by probiotic use in severely ill patients (Yelin et al., 2019), including cases of septicemia (Koyama et al., 2019), pneumonia, meningitis, and endocarditis (Pasala et al., 2020). This is of particular concern for preterm infants with immature intestinal epithelial cells who are administered probiotics to reduce the risk of necrotising enterocolitis (Dani et al., 2016). There has been a growing number of reports of probiotic-related adverse effects in newborns (Esaiassen et al., 2016). In addition, live probiotics may permanently colonize the GIT of neonates and thus prevent colonization with the indigenous microbiota (Deshpande et al., 2018). Another concern with live probiotics relates to their potential to act as a conduit for antibiotic-resistance genes, which have been detected in common probiotic species (Baumgardner et al., 2021) and commercial probiotic products (Selvin et al., 2020). While probiotics that harbor resistant genes are not inherently dangerous, the transfer of these genes to opportunistic pathogens has been demonstrated and may have serious clinical ramifications (Montassier et al., 2021). In addition to the limitations that apply to the wild-type probiotic strains, recombinant probiotics face additional hurdles on the road to clinical development. Their use in human medicine would entail a release of genetically modified organisms (GMOs) into the environment, which raises additional safety and ecological risks such as unexpected interactions with host organisms, the transfer of synthetic genes to wild species, and environmental persistence (Plavec and Berlec, 2020). It is therefore necessary for live recombinant probiotics to undergo rigorous testing to ensure that they cannot spread in the environment or transfer genetic changes to other organisms. From a regulatory perspective, recombinant probiotics fall into the category of live biotherapeutic products (Heavey et al., 2022), which are subject to stringent regulatory requirements.

To address regulatory requirements and safety issues of live probiotics, there is a growing research interest in developing non-viable probiotic bacteria (postbiotics) for therapeutic use. Postbiotics are defined as the “preparation of inanimate microorganisms and/or their components that confer a health benefit on the host” (Salminen et al., 2021). In recent years, studies have shown that postbiotics can exhibit beneficial effects on the host in many cases comparable to or even greater than those of live strains (Zhang et al., 2022). The administration of high doses of non-viable probiotic bacteria (≥1.0 × 10^10^ CFU/day) was proven to be safe (Holz et al., 2015; Andresen et al., 2020). As they are not able to replicate, the risk of bacterial infections is drastically reduced. Apart from being safer, non-viable bacteria have more favorable technological properties for manufacturing, transport, and storage. They can be formulated under conditions incompatible with microbial survival. Furthermore, the stringent regulatory requirements set up for live recombinant probiotics may not apply to postbiotics since killing treatment precludes their replication. Although the mechanisms of action of postbiotics are not completely understood, *in vitro* and *in vivo* studies show that they have anti-inflammatory, immunomodulatory, anti-proliferative, and anti-microbial activity (Cuevas-Gonzalez et al., 2020). Clinical trials have demonstrated the effectiveness of postbiotics in the management of atopic dermatitis (Jeong et al., 2020; Rather et al., 2021), respiratory allergies (Pique et al., 2019), diarrhea (Malagón-Rojas et al., 2020) and inflammatory bowel disease (IBD) (Wang et al., 2024). Examples include heat-killed *Bifidobacterium bifidum* MIMBb75, which has shown clinical efficacy in irritable bowel syndrome (Andresen et al., 2020), heat-killed *L. acidophilus* LB in treating diarrhea (Liévin-Le Moal, 2016), and heat-killed *Limosilactobacillus reuteri* DSMZ 17648 in adjuvant therapy of *Helicobacter pylori* infection (Holz et al., 2015). *L. acidophilus* LB and *Lactobacillus reuteri* DSM17648 are patented and incorporated in commercial postbiotic products (Lactéol™, Pylopass™) (Liang and Xing, 2023; Vinderola et al., 2023). In contrast to wild-type probiotics, studies on non-viable forms of recombinant probiotics are very scarce. A small number of studies have demonstrated the efficacy of heat- or formaldehyde-killed *Escherichia coli* CWG308 engineered to display oligosaccharide mimic of Shiga toxin receptor (Paton et al., 2001), and a few studies, which showed that heat-killed recombinant *Lactobacillus casei* expressing E7 mutated protein was effective in eliciting mucosal immune response against human papillomavirus type 16 (Kawana et al., 2014).

Genetically engineered lactic acid bacteria (LAB) are being developed as live biotherapeutic products for food allergies (Zahirovic and Lunder, 2018), radiation-induced mucositis (Limaye et al., 2013), and IBD (Braat et al., 2006). Several species of LAB, especially *Lactococcus lactis* (*L. lactis*), have been shown to be effective as mucosal delivery vehicles of antibody mimetics for adjunctive therapy of IBD (Vandenbroucke et al., 2010). One of the promising new strategies is the expression of cytokine-binding proteins on the surface of LAB to equip the cells with the ability to remove proinflammatory cytokines from the gut lumen and thus impede the perpetuation of the inflammatory process. The main pro-inflammatory cytokines that drive intestinal inflammation in IBD are tumor necrosis factor (TNF), interleukin (IL)6, IL12, IL23, IL17, and IFN-γ (Neurath, 2022). IL6 also plays an important role in the development of inflammation-associated colorectal cancer through hyperactivation of IL6/STAT3 pathway (Heichler et al., 2020). Elevated IL6 levels were found in serum and tumor tissue of patients with colorectal cancer (Galizia et al., 2002; Nikolaus et al., 2018). Thus, the inhibition of IL6 may not only reduce intestinal inflammation but also decrease the risk of colorectal cancer. By expressing small protein binders of proinflammatory cytokines on the bacterial surface, we constructed several recombinant strains of *L. lactis*, which show the ability to bind IL6, TNF, IL17 or IL8 in our previous studies (Ravnikar et al., 2010; Kosler et al., 2017; Skrlec et al., 2017; Zahirovic and Berlec, 2022). Protein binders included were affibody against IL6 (Zahirovic and Berlec, 2022), affibody against TNF (Ravnikar et al., 2010), IL17-targeting fynomer (Kosler et al., 2017) and IL8-targeting evasin-3 (Skrlec et al., 2017). Affibodies are derivatives of the Z domain from staphylococcal protein A composed of a characteristic three-helix scaffold (Oroujeni et al., 2024). Fynomers are derived from the SH3 domain of the human Fyn tyrosine kinase, which forms a compact β-barrel made of five β-strands connected by four loops (Skrlec et al., 2015). Evasin-3 is natural tick salivary proteins consisting of three β-strands with six conserved cysteine residues that form a disulfide-bonded knottin structure (Aryal et al., 2022). The binders were non-covalently anchored to the cell wall of *L. lactis* via lysine motif (LysM) contained in a C-terminal peptidoglycan-binding domain (cAcmA) of major lactococcal autolysin AcmA. In the present study, we prepared non-viable forms of these recombinant *L. lactis* strains by employing various physicochemical treatments and performed detailed *in vitro* characterization of their functionality in comparison with that of live counterparts. The study aims to establish whether recombinant LAB displaying protein binders on their surface can retain their function after the loss of bacterial viability and thus show postbiotic potential.

## 2 Materials and methods

### 2.1 Bacterial strains, growth conditions and protein expression

The bacterial strain and plasmids used in this study are listed in Table 1. The plasmids for surface display of cytokine binders were previously constructed by cloning in pNZ8148 the genes for respective cytokine-binding protein, each fused to lactococcal secretion signal Usp45 and anchoring domain cAcmA as described in (Ravnikar et al., 2010; Kosler et al., 2017; Skrlec et al., 2017; Zahirovic and Berlec, 2022). The constructed plasmids were transformed into *Lactococcus lactis* NZ9000 (recently renamed *Lactococcus cremoris* NZ9000) by electroporation using Gene Pulser II (Biorad, Hercules, CA, USA). For protein expression, overnight cultures of the recombinant *L. lactis* strains harboring the appropriate plasmids were diluted 1:50 in M17 medium (Merck, Darmstadt, Germany) supplemented with 0.5% glucose and 10 μg/mL of chloramphenicol. Bacteria were grown at 30 °C without shaking until they reached an early exponential growth phase (OD_600_ between 0.6 and 0.8) when nisin (10 ng/mL, Sigma) was added to induce protein expression. After 3 h of incubation, the cells were harvested by centrifugation (5,000 × g for 10 min at 4 °C) and resuspended in phosphate-buffered saline (PBS). OD_600_ was adjusted to 1.0, which was determined to correspond to 1 × 10^8^ CFU/mL by culturing and enumeration of colony-forming units (CFUs). CFU counting was performed using a drop plate method by pipetting 10 μl drops of 10-fold serial dilutions of bacteria on agar plates. Following incubation at 30 °C for 48 h, colonies were counted and CFU/mL was calculated.

**Table 1:** The bacterial strain and plasmids used in this study.

**Strain or plasmid**	**Relevant features**	**Reference or source**
**Strain**		
*L. lactis* subsp. *cremoris* NZ9000	MG1363 *nisRK* Δ*pepN*	NIZO
**Plasmids**		
pNZ8148	pSH71 derivative, P*_*nisA*_*, Cm^r^, nisin-controlled expression	(Mierau et al., 2005)
pSD-ZIL6	pNZ8148 containing gene fusion of *sp_*Usp*45_, flag, zil* and *acmA3b*	(Zahirovic and Berlec, 2022)
pSD-ZTNF	pNZ8148 containing gene fusion of *sp_*Usp*45_, ztnf* and *acmA3b*	(Ravnikar et al., 2010)
pSD-Fyn17	pNZ8148 containing gene fusion of *sp_*Usp*45_, fyn17* and *acmA3b*	(Kosler et al., 2017)
pSD-EVA	pNZ8148 containing gene fusion of *sp_*Usp*45_, eva-3* and *acmA3b*	(Skrlec et al., 2017)

NIZO food research BV (the Netherlands).

### 2.2 Treatments of recombinant *L. lactis* for generation of postbiotics

To generate non-viable *L. lactis* cells, several different physicochemical treatments were applied, including exposure to heat, sonication, ethanol, UV, and gamma irradiation. The treatments were carried out using 1 mL aliquots of bacterial suspension in PBS in 1.5 mL polypropylene microcentrifuge tubes at a concentration of 1 × 10^8^ CFU/mL (OD 1). Bacteria were heat-treated in a water bath at 70 °C for 40 min or at 100 °C for 30 min. Sonication was performed using a UPS200S sonicator (Hielscher, Teltow, Germany) operating at a frequency of 20 kHz and output power of 10 W. Using a 1 mm-diameter probe, the samples were sonicated in two cycles lasting 15 s each, followed by incubation on ice to avoid excessive heating. Ethanol treatment was performed by suspending bacterial cells in 70% ethanol and incubating for 5 min at room temperature. For UV irradiation, open microcentrifuge tubes with bacteria suspensions were placed in a laminar flow cabinet at a distance of 80 cm from a germicidal UV-C lamp (15 W, 254 nm) for 30 min. This lamp typically provides a UV intensity of approximately 44 μW/cm^2^ at 1 meter distance. Gamma irradiation was carried out at the JSI TRIGa Mark II research reactor (JoŽef Stefan Institute, Slovenia) by utilizing the delayed gamma rays from irradiated fuel (AmbroŽič et al., 2018; AmbroŽič and Snoj, 2020). The total accumulated gamma ray dose was 1 kGy with the dose rate of ~ 50 Gy/h, ~ 200 Gy/h, or ~ 270 Gy/h.

Following treatments, bacteria were centrifuged at 5,000 × *g* for 10 min. The concentration of extracellular DNA in the supernatants was determined by measuring the absorbance at 260 nm using NanoDrop ND-2000c (Thermofisher Scientific, Walthman, MA, USA). Bacterial cells were washed once with PBS (ethanol-treated bacteria were washed twice to remove any residual traces of ethanol), adjusted to the appropriate OD_600_, and used for analyses. The bacteria were stored at 4 °C, and the growth was determined 1 day after the treatment and after 14 days of storage.

### 2.3 ELISA assay for assessment of cytokine binding by bacteria

Removal of cytokines from the solution by the treated and non-treated recombinant *L. lactis* was analyzed by enzyme-linked immunosorbent assay (ELISA) as previously described (Zahirovic et al., 2022). Briefly, cytokine standards IL6, TNF, IL17, or IL8 (300 pg/mL; Mabtech, Nacka Strand, Sweden) were incubated with 1 × 10^8^ CFU/mL of the respective recombinant bacterial strain. Since anti-IL8 evasin-displaying *L. lactis* was weaker binder than the other three strains, these bacteria were used at three times higher concentration (3 × 10^8^ CFU/mL), which has been reported to exhibit considerable cytokine binding in our previous studies (Skrlec et al., 2017; Zahirovic et al., 2022). Incubation was carried out in 650 μl buffered saline solution (PBS containing 0.05% Tween 20 and 0.1% bovine serum albumin) for 2 h at room temperature with gentle agitation. The samples were then centrifuged to remove bacterial cells, and the remaining amount of cytokine in the supernatant was quantified using ELISA Flex Human IL6, TNF, IL17 or IL8 (HRP) kit (Mabtech) by following the protocol in (Zahirovic et al., 2022). The percentage of bound cytokines was calculated as the difference between the amount of cytokine that remained after incubation with binder-displaying bacteria and the amount that remained after incubation with the control bacteria (carrying empty plasmid pNZ8148).

### 2.4 SYTO 9/PI staining

To determine cell membrane integrity upon treatments, bacteria were stained with SYTO 9 (S34854, Invitrogen, Carlsbad, CA, USA) and propidium iodide (PI, P3566, Invitrogen, Walthman, MA, USA). Staining was performed by adding the dyes directly to bacterial suspension (2.7 × 10^7^ CFU/mL) to a final concentration of 1 μM SYTO 9 and 15 μM PI. The samples were vortexed and then incubated for 15 min at room temperature, protected from light. Stained bacteria were immobilized on glass microscope slides coated with poly-L-lysine 0.01% solution (A-005-C, Merck) using StatSpin Cytofuge2 (Beckman Coulter, Brea, CA, USA). The samples were mounted with Prolong^TM^ Gold Antifade Mountant (P36930, Invitrogen). Bacteria were visualized with a Zeiss LSM 710 laser scanning confocal microscope (Carl Zeiss, Jena, Germany) using a Plan-Apochromat 63 × /1.4 NA oil objective lens. Images were acquired in sequential acquisition mode. The SYTO 9 was first excited by a 488 nm laser, and the emission light of SYTO 9 was collected in the 493–565 nm spectral window, followed by the excitation of the PI by a 543 nm laser and collection of the emission light of PI in the 566–719 nm spectral window. The cells were also stained with each dye separately and examined under the same filter sets. No bleedthrough of the SYTO 9 emission into the red channel or the PI emission in the green channel was observed. The images were processed using Image J 1.52g (Schneider et al., 2012).

### 2.5 Resazurin assay

For evaluation of metabolic activity, bacterial suspensions of treated or non-treated *L. lactis* were pipetted into a black 96-well plate (180 μl/well) at different concentrations (2-fold dilutions in PBS ranging from 6 × 10^6^ to 1 × 10^8^ CFU/mL) and 20 μl of 0.4 mg/mL resazurin solution (R7017, Merck) was added to each well. The samples were subsequently incubated at 37 °C for 24 h. The fluorescence was recorded with a microplate reader (Infinite M1000, Tecan, Austria) at the excitation of 550 nm and emission of 590 nm.

### 2.6 Scanning electron microscopy (SEM) analysis of bacteria

Scanning electron microscopy (SEM; Supra35 VP, Carl Zeiss) was used to analyse bacterial morphology (size, shape, and surface structure) upon treatments. As a positive control, *L. lactis* bacteria with destroyed cell structure were obtained by enzymatic lysis with 2 mg/mL lysozyme and 100 UI mutanolysin (30 min incubation at 37 °C). Bacteria were washed four times in sterile distilled water and then pipetted on the metal stubs at different dilutions (four 2-fold dilutions of bacterial samples with an initial concentration of 1 × 10^9^ CFU/mL). The morphology of individual cells without sputter coating was visualized under different magnifications at an accelerating voltage of 1 kV, using a secondary detector. The size of 50 individual bacterial cells was measured for each treatment using ImageJ 1.52g (Schneider et al., 2012).

### 2.7 Characterization of ZIL6 affibody surface display on bacteria by fluorescence microscopy and flow cytometry

To compare the surface display of ZIL6 affibody between treated and non-treated cells, bacteria (8 × 10^6^ CFU) were probed with anti-flag antibodies directed toward flag-tagged affibody on the cell surface. Labeled bacteria were analyzed by fluorescence microscopy and flow cytometry as previously described (Zahirovic and Berlec, 2022). Immunolabeling was carried out according to the protocol published in (Plavec et al., 2021b). For affibody labeling, we used rabbit anti-flag primary antibodies (20543-1-AP, Proteintech) and goat anti-rabbit secondary antibodies conjugated to Alexa Fluor 488 (for flow cytometry) or Alexa Fluor 555 (for microscopy) (4412; 4413, Cell Signaling Technology, Danvers, MA, USA). FM 1-43 dye (T3163, Thermofisher Scientific) was used for staining cell membranes at a final concentration of 0.5 μM. The images were acquired with a Zeiss LSM 710 laser scanning confocal microscope (Carl Zeiss). Deconvolution of fluorescence microscopy images was performed using the DeconvolutionLab2 plugin in Image J (version 1.52p) (Schneider et al., 2012). The geometric mean fluorescence intensity (MFI) of the population of 20,000 bacterial cells was measured with a flow cytometer (FACS Calibur; BD Biosciences, Franklin Lakes, NJ, USA). The samples were excited by the 488 nm laser, and the emission fluorescence was acquired in the FL1 channel (530/30 nm). Flow cytometric data were analyzed and displayed with the FlowJo v10.8 software (BD Biosciences).

### 2.8 Quantitative assessment of bacterial binding affinity by flow cytometry and maximum binding capacity by ELISA

A time-course experiment was conducted to determine the incubation time required to reached equilibrium in the binding of IL6 to ZIL6-displaying *L. lactis*. Bacteria (1 × 10^8^ CFU/mL) were incubated with recombinant human IL6 (1,000 pg/mL; HZ-1019; Proteintech) for various time intervals (10, 20, 30, 40, 60, 90, 120, 150, 160, 180 min). Following incubation, the remaining IL6 in the supernatant was quantified for each time point by ELISA according to the protocol in (Zahirovic et al., 2022).

The binding affinity of bacteria for IL6 was evaluated by flow cytometry according to the protocol published in (Plavec et al., 2021a). Briefly, serial dilutions of IL6 (0.32, 0.65, 1.31, 2.62, 5.25, 10.5, 21, 42 nM; HZ-1019, Proteintech) were incubated with a fixed number of recombinant bacteria (8 × 10^6^ CFU) for 2 h in a final volume of 200 μl. Bacteria-bound IL6 was detected with rabbit anti-human IL6 polyclonal antibodies (21865-1-AP, Proteintech) and goat anti-rabbit Alexa Fluor 488-conjugated secondary antibodies (4412, Cell Signaling Technologies). The amounts of IL6 bound to the bacteria correlate with the mean fluorescence intensities measured in the population of 20 000 bacterial cells with a flow cytometer (FACS Calibur; BD Biosciences). Equilibrium dissociation constants (Kd) were determined using non-linear regression (Hill equation) in GraphPad Prism v.10.3.1 (GraphPad Software Inc., USA).

To quantify the maximum binding capacity of bacteria, the increasing amounts of IL6 (0.45, 4.5, 45 and 450 ng; HZ-1019, Proteintech) were spiked into 450 μl buffered saline solution (PBS containing 0.05% Tween 20 and 0.1% bovine serum albumin) and incubated with a constant number of ZIL6-displaying *L. lactis* (4.5 × 10^7^ CFU) for 2 h. The residual IL6 in the supernatant was determined by ELISA according to the protocol in (Zahirovic et al., 2022). The supernatants were appropriately diluted to fall within the linear range of the standard curve (10–1,000 pg/mL). The amount of bound IL6 per mg dry cell weight of bacteria was calculated by the following equation: Bound amount (ng/mg) = (C_0_-C_f_) x V/m, where C_0_ and C_f_ are concentrations of IL6 before and after incubation with bacteria, respectively (ng/mL), V is final incubation volume (mL) and m is dry cell mass of bacteria (mg).

### 2.9 HEKblue-IL6R cell assay

The biological activity of ZIL6-displaying *L. lactis* was examined by monitoring the inhibition of IL6-induced STAT3 signaling in HEKblue-IL6R reporter cell line. HEKblue-IL6R cells (hkb-hil6, InvivoGen, Toulouse, France) are generated by stable transfection of human embryonic kidney HEK293 cell line with cDNA encoding human IL6 receptor (IL6R), the signal transducer and transcription activator 3 (STAT3), and a secreted embryonic alkaline phosphatase (SEAP). The binding of IL6 to the receptor activates the JAK/STAT3 pathway, leading to the secretion of SEAP that is measured in cell culture supernatant. HEKblue-IL6R cells were grown in DMEM medium (Gibco, Life Technologies) supplemented with 10% heat-inactivated fetal bovine serum (FBS, Gibco), 100 U/mL penicillin, 100 μg/mL streptomycin (Gibco), 100 μg/mL Normocin (InvivoGen), and 1 × HEK-Blue™ Selection (InvivoGen). The assay was carried out according to the manufacturer's instructions. First, a dose-response experiment was performed by stimulating HEKblue-IL6R cells (50 000 cells/well in a 96-well plate) with increasing concentrations (10-fold dilutions ranging from 0.001 to 100 ng/mL) of recombinant human IL6 (3460-1H-6, Mabtech) in 180 μL DMEM medium supplemented with 10% heat-inactivated FBS. After 24 h incubation, the SEAP-containing culture supernatants (20 μL/well) were collected and incubated with Quanti-Blue substrate (180 μL/well) for 1-2 h. The absorbance was recorded at 630 nm with a microplate reader (Infinite M1000, Tecan). Unstimulated HEKblue-IL6R cells or HEKblue-IL6R cells stimulated with cytokines that do not signal through the JAK/STAT3 pathway, recombinant human TNF (1 ng/mL; 3512-1H-6, Mabtech) or recombinant human IL8 (1 ng/mL; 3560-1H-6, Mabtech), were used as negative controls. For inhibition assay, bacteria (1 × 10^8^ or 1 × 10^9^ CFU/mL) were preincubated with IL6 (1 ng/mL; 3460-1H-6, Mabtech) in 100 μL DMEM medium supplemented with 10% heat-inactivated FBS for 2 h at room temperature under mild agitation. Bacterial cells were then pelleted by centrifugation (7,500 × g, 7 min), and supernatants (20 μL/well) containing the remainder of IL6 were used to stimulate the HEKblue-IL6R cells. The amount of SEAP secreted into the cell medium was detected as described above for dose-response experiment. The percentage of STAT3 inhibition was calculated relative to the STAT3 signaling induced by IL6 in the absence of bacteria. The inhibition ability of bacteria was compared to that of anti-IL6 monoclonal antibody (3460-1H-6, Mabtech). To test the influence of bacteria on cell viability, HEKblue-IL6R cells (100 000 cells/well) were incubated in a 96-well plate with treated or non-treated *L. lactis* (2 × 10^7^ CFU/well) for 6, 12 and 24 h, and the viability was determined with trypan blue (T8154, Merck).

### 2.10 Statistical analysis

Statistical analysis was performed with GraphPad Prism v.10.3.1 (GraphPad Software Inc., USA). Unpaired Student's *t*-test was used to determine a statistically significant difference between the binder-displaying *L. lactis* and control *L. lactis* cells (carrying empty plasmid pNZ8148). Statistically significant differences between the treated and non-treated bacteria were determined using a one-way analysis of variance (ANOVA) followed by a Dunnett *post-hoc* multiple comparison test. Results were considered significant at *p* < 0.05.

## 3 Results

### 3.1 Recombinant *L. lactis* bacteria are not able to grow on agar plates after exposure to heat, ethanol, and gamma irradiation

**Figure 1:**
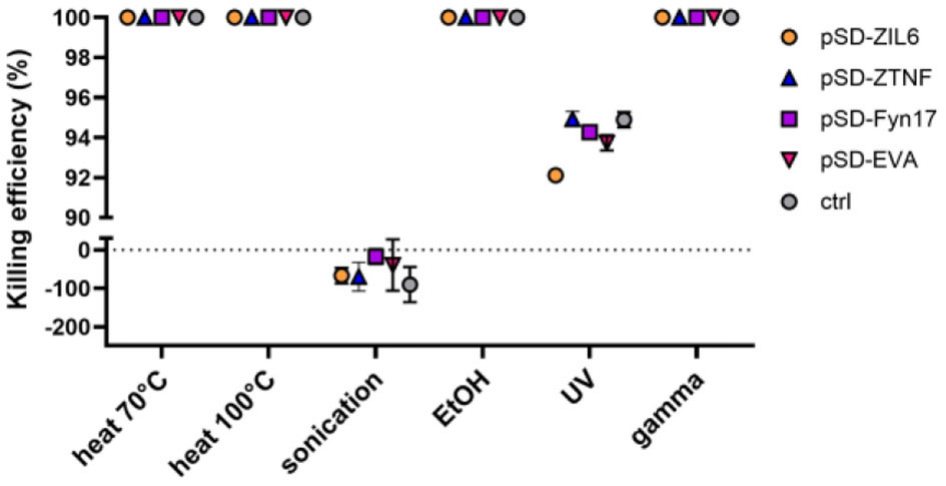
Killing efficiency of different physicochemical treatments on recombinant *L. lactis* strains displaying different binders of proinflammatory cytokines on their surface. The killing efficiency was determined by culturing and counting colonies on agar plates. Complete killing of bacteria i.e., 0 CFU/mL (no bacterial colonies on agar plates) was considered as 100% efficiency. pSD-ZIL6, *L. lactis* displaying anti-IL6 affibody; pSD-ZTNF, *L. lactis* displaying anti-TNF affibody; pSD-Fyn17, *L. lactis* displaying anti-IL17 fynomer; pSD-EVA, *L. lactis* displaying anti-IL8 evasin; pNZ8148, *L. lactis* harboring empty plasmid. The results presented are means ± standard deviation (SD) of three technical replicates from a representative experiment.

To determine which method is most suitable for generation of postbiotics from recombinant *L. lactis*, a suspension of stationary-phase bacteria was subjected to a treatment with heat (at 70 °C for 40 min or 100 °C for 30 min), sonication, ethanol, UV, or gamma irradiation. The killing efficiency of the treatments was determined by culturing and counting colonies on agar plates. Under the conditions applied in this study, the treatment with heat, ethanol and gamma irradiation caused complete killing of bacteria (the cells were incapable of growth on agar plates), while UV irradiation was not entirely effective and killed 91.2–94.5% of cells (Figure 1). Following sonication, CFU counts increased (by 17% to 90%) in all tested bacterial strains, which is attributed to the disintegration of lactococcal chains into single cells. The growth of bacteria on solid and liquid media was also tested after a 14-day storage period at 4 °C. Non-viable bacteria did not recover the growth, indicating that the damage could not be repaired (Supplementary Figure 1).

### 3.2 The effect of the treatments on cytokine-binding ability of recombinant *L. lactis* bacteria depends on the type of binder displayed on their surface

**Figure 2:**
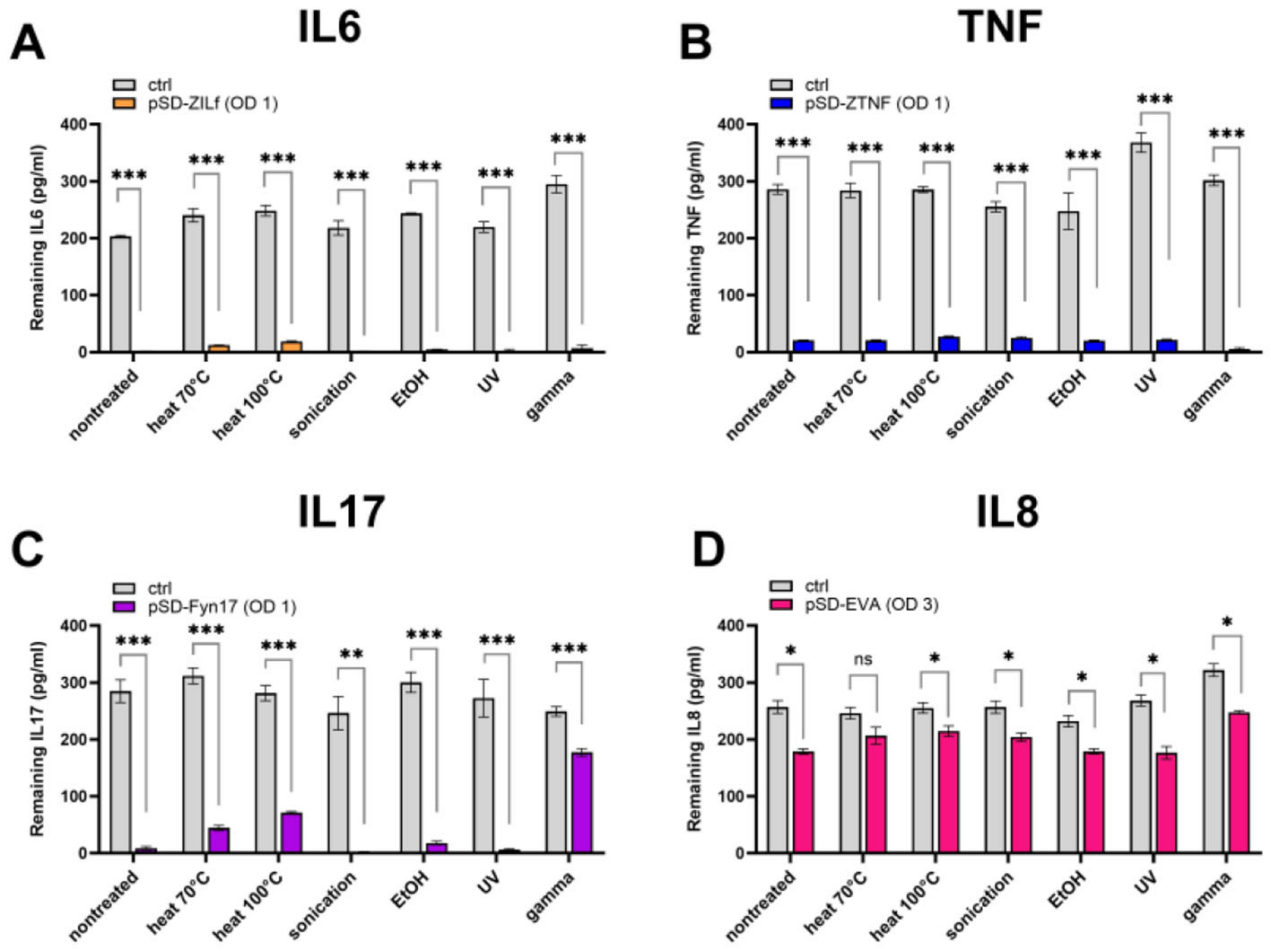
Cytokine binding ability of recombinant *L. lactis* bacteria displaying different binders of proinflammatory cytokines on their surface upon exposure to bacteria-killing treatments. ELISA-determined concentrations of recombinant IL6 **(A)**, TNF **(B)**, IL17 **(C)**, and IL8 **(D)** that remained in the solution following incubation with the corresponding strain of bacteria before and after treatment with heat (70 °C, 40 min or 100 °C, 30 min), sonication, ethanol, UV, and gamma irradiation. pSD-ZIL6, *L. lactis* displaying ZIL6 affibody; pSD-ZTNF, *L. lactis* displaying anti-TNF affibody; pSD-Fyn17, *L. lactis* displaying anti-IL17 fynomer; pSD-EVA, *L. lactis* displaying anti-IL8 evasin; Ctrl: *L. lactis* control cells containing empty plasmid pNZ8148. The results are presented as means ± SD of three technical replicates of a representative experiment. ns, *p* = 0.09; *, *p* ≤ 0.05; **, *p* = 0.002; ***, *p* < 0.001 (unpaired two-tailed *t*-test).

To assess whether the surface-displayed cytokine binders can maintain their functionality upon treatments, the cytokine binding ability of four bacterial strains was evaluated in a preliminary ELISA experiment at a cytokine concentration of 300 pg/mL. Recombinant bacteria displaying binders with different scaffolds (affibody, fynomer, evasin) were tested to find out which is least affected by the treatments and select the strain for further characterization. Our results show that the strains were affected to a varying degree depending on the type of binder on their surface (Figure 2). Affibodies appear to be unaffected by the treatments, whereby the cytokine binding ability of treated affibody-displaying bacteria (both ZIL6 affibody- and ZTNF affibody-displaying strain) was comparable to that of non-treated cells (Figures 2A, B). In contrast, anti-IL17 fynomer-displaying strain was adversely affected by exposure to heat at 100 °C and gamma irradiation, resulting in a 20% and a 64% reduction of binding ability, respectively (Figure 2C). Even greater negative effect was observed for anti-IL8 evasin-displaying strain, which was negatively impacted by most of the treatments with a 24, 32, 36, 48, and 49% reduction in binding ability after exposure to ethanol, sonication, gamma irradiation, and heat treatment (at 70 °C or 100 °C), respectively (Figure 2D). Based on these results, we selected one of the affibody-displaying strain, ZIL6-displaying *L. lactis*—specifically targeting IL6—for detailed characterization of morphological features and engineered functionality of treated cells in order to evaluate their postbiotic potential.

### 3.3 Heat- and ethanol treatment cause cell membrane damage and inhibition of metabolic activity in ZIL6-displaying *L. lactis* bacteria

**Figure 3:**
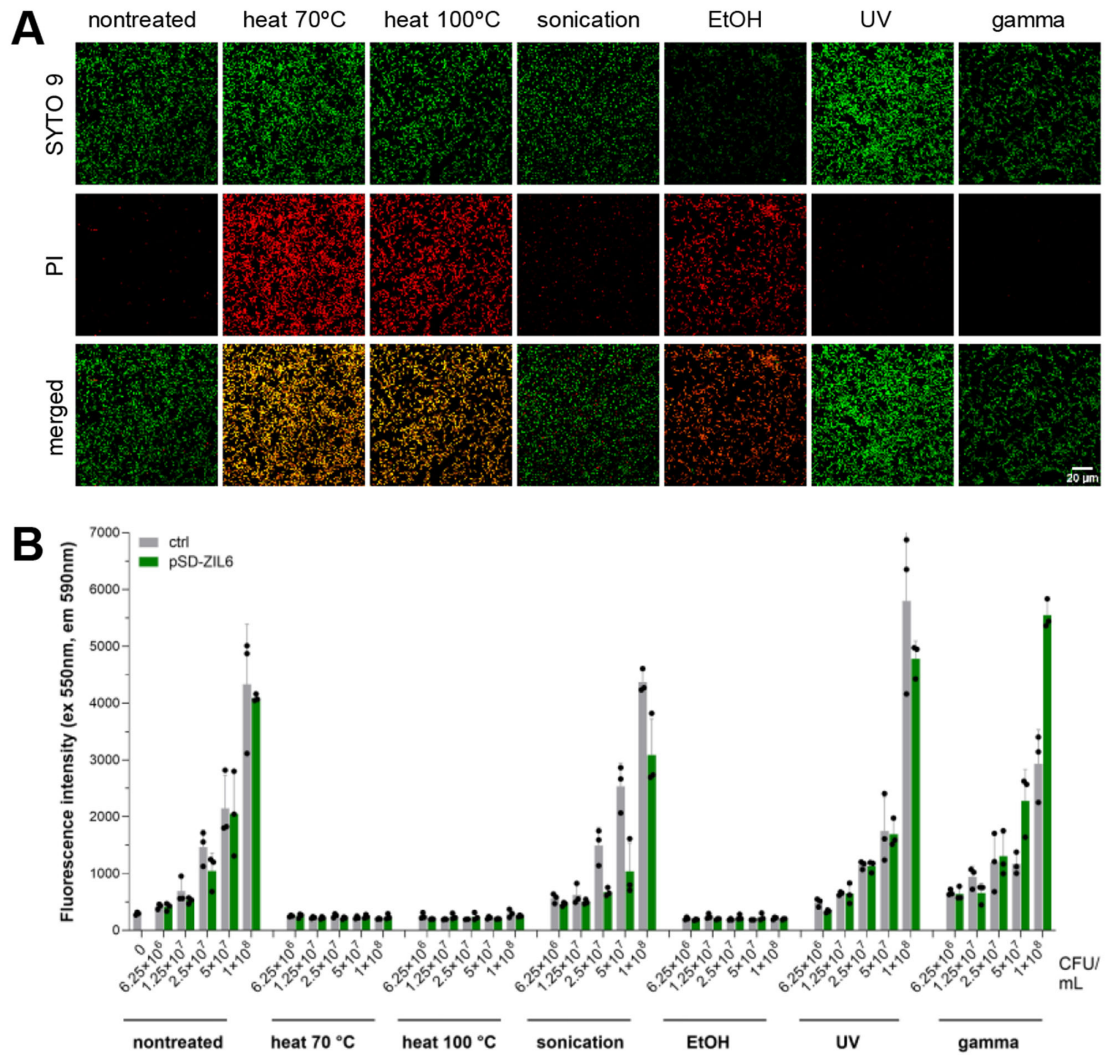
**(A)** Cell membrane integrity of ZIL6-displaying *L. lactis* bacteria upon treatments. Fluorescent microscopy images of SYTO 9 and PI stained ZIL6-displaying *L. lactis* before and after treatment with heat (70 °C, 40 min or 100 °C, 30 min), sonication, ethanol, UV, and gamma irradiation. In the merged images, the cells with intact membranes are stained green (SYTO 9^+^PI^−^), the cells with damaged membranes are stained red (SYTO 9^−^PI^+^), whereas the cells with a partially disrupted membrane are stained orange (SYTO 9^+^PI^+^). **(B)** Metabolic activity of recombinant *L. lactis* bacteria upon treatments. Resazurin assay of ZIL6-displaying *L. lactis* before and after each treatment. Data represents the mean ± SD of three technical replicates of a representative experiment. pSD-ZIL6, *L. lactis* displaying ZIL6 affibody; Ctrl: *L. lactis* control cells containing empty plasmid pNZ8148.

Bacterial membrane integrity was assessed by SYTO 9/PI staining based on the difference between these two dyes in membrane penetrability and affinity for DNA. The membrane-permeant dye SYTO 9 enters all cells, while PI can enter only membrane-damaged cells. When DNA is exposed to both dyes, PI displaces SYTO 9 due to its higher affinity for DNA. As shown in Figure 3A, the membrane integrity of recombinant *L. lactis* was preserved following sonication, UV, and gamma irradiation (negative PI staining), whereas cell membrane integrity was disrupted in bacteria treated with heat and ethanol (positive PI staining). After counterstaining, the SYTO 9 signal was present in heat-killed cells, along with PI. As previously reported, incomplete displacement of PI by SYTO 9 can occur due to more intense SYTO 9 staining of dead cells compared to live cells (Stiefel et al., 2015). When we compared the fluorescence intensity of SYTO 9 single-staining between live and non-viable *L. lactis* considerably higher SYTO 9 single staining can be seen in heat-killed bacteria, which likely contributes to the green signal in these cells after counterstaining with PI (Supplementary Figure 2). This demonstrates that heat treatment increases membrane permeability of *L. lactis* for SYTO 9. Metabolic activity of bacteria upon the treatments was determined with resazurin assay based on the resazurin reduction by oxidoreductases in the cells with active metabolic processes. The results corroborated with SYTO 9/PI co-staining and showed that the bacteria with intact cell membrane (sonicated, UV- and gamma-irradiated cells) preserved their metabolic activity, whereas the cells with damaged cell membrane (heat- and ethanol-killed bacteria) lacked metabolic activity (Figure 3B). These results illustrate that gamma-irradiated cells and the majority (91%) of UV-irradiated bacteria have lost culturability, while the cell membrane and the oxidoreductase (resazurin reduction) activity remained intact.

### 3.4 Cellular integrity is maintained in all treated cells with small alterations in surface structure

**Figure 4:**
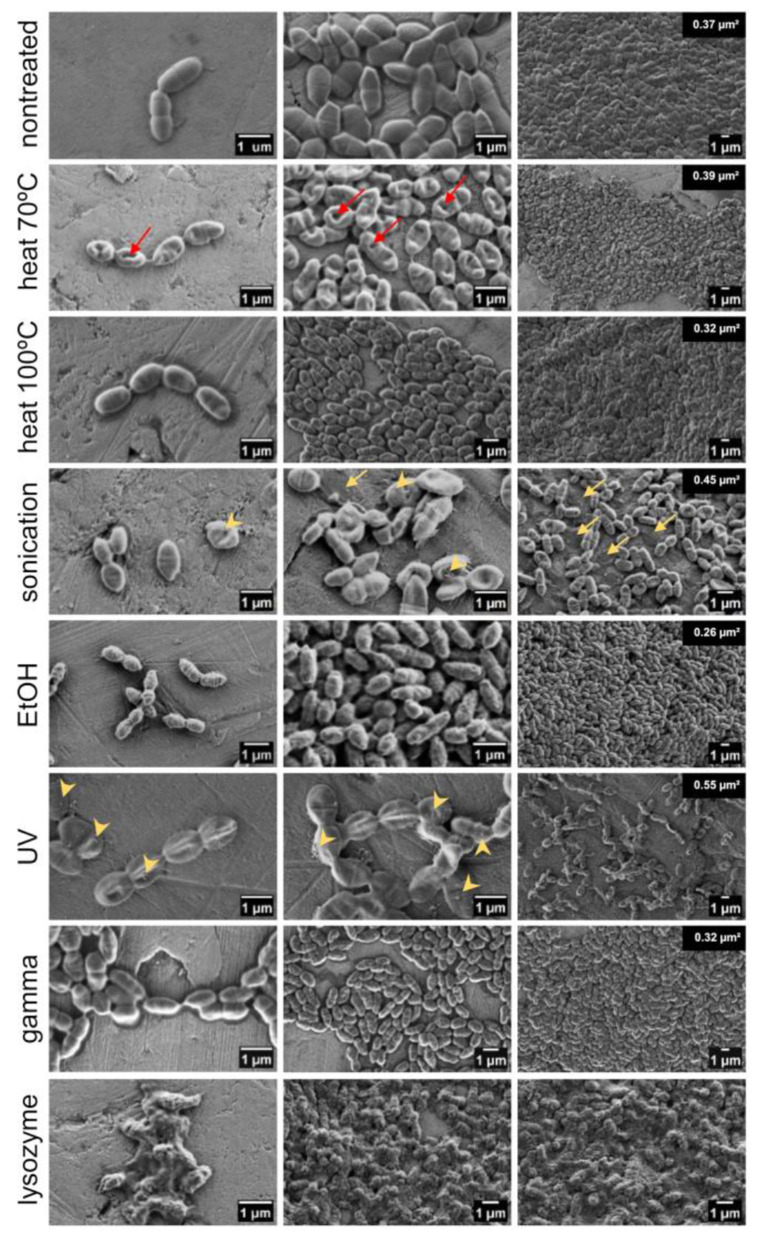
Morphology and surface structure of ZIL6-displaying *L. lactis* bacteria before and after treatments. Representative scanning electron micrographs of non-treated *L. lactis* cells compared to the bacteria exposed to heat (70 °C, 40 min or 100 °C, 30 min), sonication, ethanol, UV, gamma irradiation, and lysozyme/mutanolysin treatment. Bacterial samples were air dried from the water suspension (image depicts the cells under high, medium and low magnification). Red arrows indicate dents in the surface of the cells subjected to heat at 70 °C, while the yellow arrows indicate deflated cells after sonication. The extracellular surface structures on sonicated and UV-treated bacteria are marked by yellow arrowheads. The numbers on the top right of the rightmost images indicate the average bacterial cell area quantified using ImageJ.

To determine whether the cell integrity of bacteria was retained after applied treatments, bacterial morphology (size, shape, and surface structure) was visualized by SEM. Treatment with lysozyme and mutanolysin was included as a positive control of cell wall degradation and destruction of cellular integrity. It caused complete cell collapse with released DNA, cell debris, and clumping of intracellular material into big aggregates. Conversely, the cell integrity of all other treated bacteria was largely preserved with minor changes in the cell size and surface structure. While non-treated live bacteria had smooth surfaces, UV- and gamma-irradiated cells displayed slight increase in surface roughness, ethanol-killed cells were wrinkled or dehydrated, and bacteria subjected to heat (at 70 °C) had indentations in the cell surface (Figure 4). Following sonication, the morphology of a small fraction of bacteria was altered, with some cells appearing as empty deflated shells, which is characteristic of cell lysis. Additionally, flocks of extracellular material can be seen in sonicated and UV-treated bacteria, suggesting a certain degree of released intracellular content or aggregated surface components. Regarding the impact of treatments on the cell size, ethanol, gamma irradiation and heat exposure (at 100 °C) shrank the cells by 30, 15, and 14%, respectively (Supplementary Figure 3). In contrast, sonication and UV treatment caused a 20% and 49% increase in cell size, respectively. The evaporation of water from the cells during heat treatment may cause cell shrinkage and indentations. The preservation of cellular integrity was confirmed by measuring extracellular DNA in the bacterial supernatants upon treatment. There was no increase in the amount of extracellular DNA in the supernatants of treated bacteria compared to the non-treated samples, thus demonstrating that DNA was contained within the cells (Supplementary Figure 4). This is supported by previous studies which found that heat- and ethanol-treated *L. lactis* retained DAPI stain (Taddese et al., 2021). The exception was a small increase in extracellular DNA in the supernatant of sonicated bacteria.

### 3.5 Ethanol treatment increases ZIL6 affibody surface display

**Figure 5:**
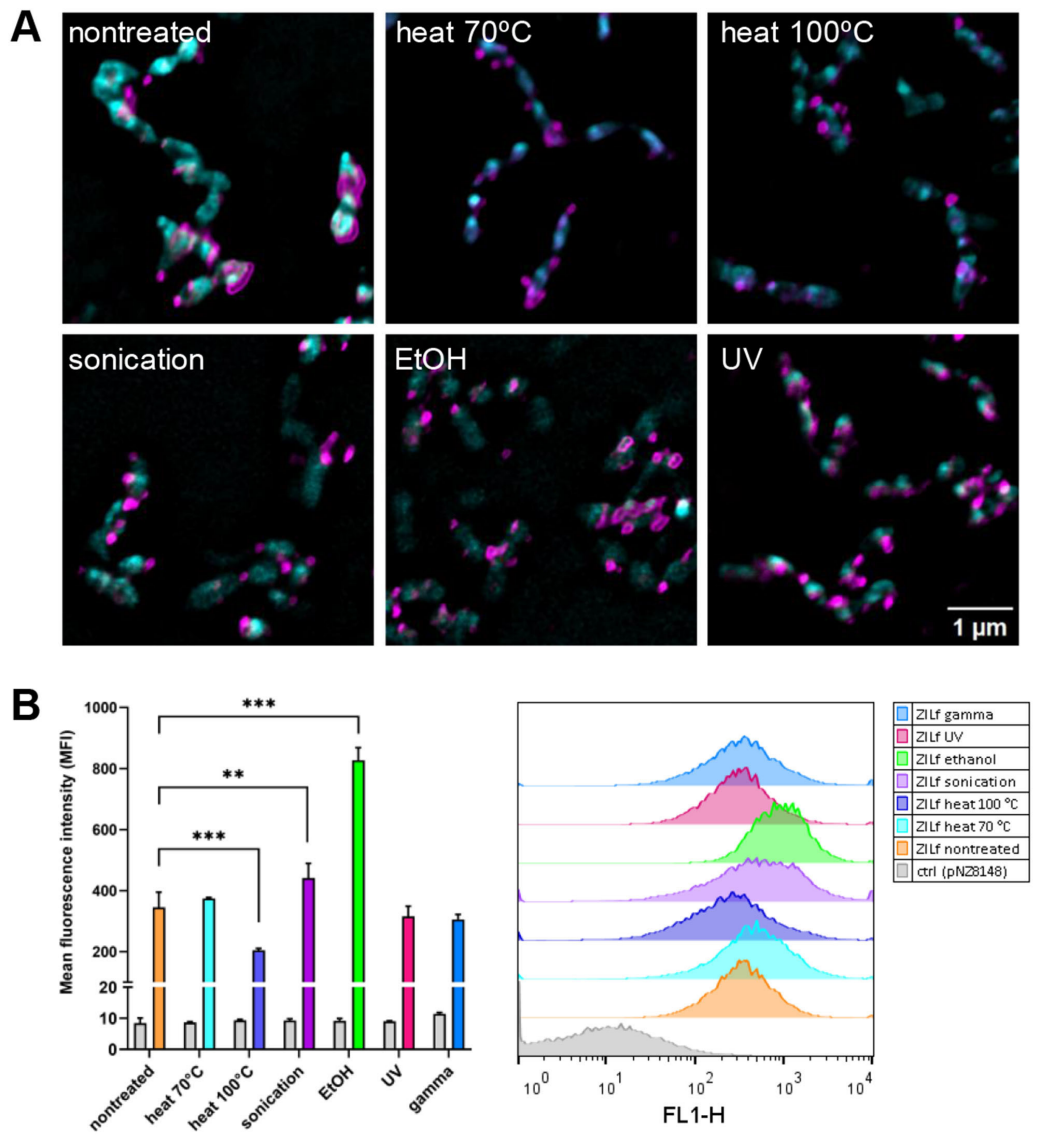
The distribution and the extent of surface display of ZIL6 affibody on non-viable recombinant *L. lactis* in comparison to the non-treated, live cells. **(A)** Representative confocal fluorescence microscopy images showing localization and distribution of ZIL6 affibody (magenta) on the surface of *L. lactis* (cyan) before and after treatment with heat (70 °C, 40 min or 100 °C, 30 min), sonication, ethanol, and UV irradiation. Bacterial cell membranes were stained with FM 1-43 lipophilic membrane probe. ZIL6 affibody was labeled with anti-flag primary antibody and Alexa Fluor 488-conjugated secondary antibody (for flow cytometry) or Alexa Fluor 555-conjugated secondary antibody (for microscopy). **(B)** Flow cytometric measurements of mean fluorescence intensities (MFI) in the population of 20 000 bacterial cells and representative intensity histograms for each treatment are depicted. The data are means ± SD of three technical replicates from a representative experiments. ZILf, *L. lactis* displaying ZIL6 affibody. Ctrl: *L. lactis* control cells harboring empty plasmid pNZ8148. **, *p* = 0.004; ***, *p* < 0.001 (one-way ANOVA with Dunnett multiple comparison test).

To determine the extent and distribution of ZIL6 affibody on the surface of *L. lactis* after treatments, the bacteria were probed with anti-flag antibodies and analyzed by fluorescence microscopy and flow cytometry. Confocal fluorescence microscopy enabled visualization of fluorescent staining at the single cell level, while flow cytometry was used to quantify the MFI in the bacterial population (of 20 000 cells). Immunofluorescence microscopy confirmed surface localization of ZIL6 affibody, with strong fluorescent signal at discrete spots on the bacterial surface, suggesting uneven distribution (Figure 5A). This is in line with previous studies, where anchoring of AcmA-fused proteins was either restricted to distinct locations (e.g., septum, poles) or distributed uniformly across the entire cell surface depending on the bacterial species and type of displayed protein (Steen et al., 2003). After the treatments, the ZIL6 affibody was retained on the cell surface, and the pattern of distribution was unchanged. The quantification of the fluorescence signal by flow cytometry shows that, in comparison to non-treated live cells, the MFI values were 41% lower in the cells killed with heat at 100 °C, indicating that the affibodies were partially stripped from these cells (Figure 5B). Conversely, the MFI for sonicated and ethanol-killed cells were higher by 28% and 140%, respectively. More than twofold increase in surface display of affibody in ethanol-killed bacteria is likely due to the removal of shielding molecules (cell membrane-tethered lipoteichoic acids, cell wall-tethered teichoic acids, or other peptidoglycan-associated polymers) from the bacterial surface by the ethanol. Importantly, ethanol-treated control cells (transformed with empty plasmid) did not fluoresce, excluding the possibility that the observed signal increase in ZIL6-displaying bacteria is caused by non-specific antibody binding to irrelevant epitopes that may have been exposed by the ethanol treatment. Slightly higher affibody surface display in sonicated bacteria may be attributed to the better accessibility of its binding sites for antibodies after the disintegration of the cell strings into individual cells. The MFI of UV- and gamma-irradiated cells as well as the cells killed by heat at 70 °C were comparable to non-treated bacteria. No binding of antibodies to control cells (carrying empty plasmid pNZ8148) was observed.

### 3.6 ZIL6-displaying *L. lactis* bacteria have nanomolar affinity for IL6, comparable between non-viable and live cells

**Figure 6:**
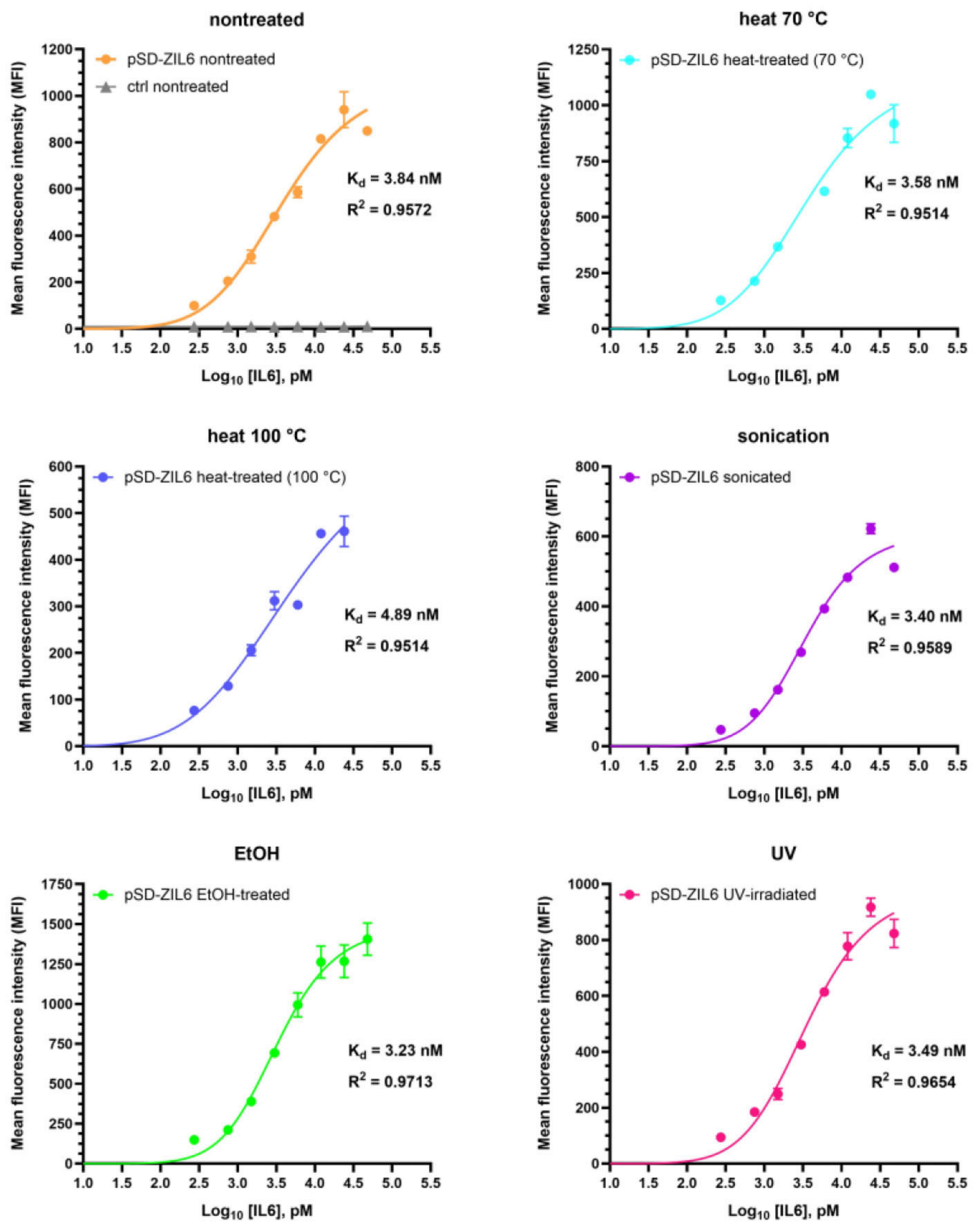
Determination of binding affinity of ZIL6-displaying *L. lactis* for human IL6. A constant number of 8 × 10^6^ CFU bacteria was incubated with increasing concentrations of human IL6 for 2 h. MFI values were measured by flow cytometry and binding curves were fitted to the data using the Hill equation in the GraphPad Prism v.10.3.1. The dissociation constants (Kd) values were calculated from the binding curves for non-treated, live ZIL6-displaying *L. lactis* bacteria and bacteria treated by heat (70 °C, 40 min or 100 °C, 30 min), sonication, ethanol, and UV irradiation. Data are means ± SD of three technical replicates from a representative experiment.

A time-course experiment showed that the binding of IL6 to ZIL6-displaying *L. lactis* was rapid, and a plateau was reached already after 10 min (Supplementary Figure 5). Continuing the incubation up to 3 h did not change the amount of protein bound. Therefore, a 2-h incubation was used in the following experiments, thus ensuring that the equilibrium is reached. Binding affinity of ZIL6-displaying *L. lactis* for IL6 was evaluated by incubating varying concentrations of IL6 (ranging from 0.32 nM to 42 nM) with a constant number of bacteria and measuring MFI of bacteria with flow cytometry. MFI values were plotted against log-transformed concentrations of IL6, yielding sigmoidal curves (Figure 6). The Hill equation was fitted to the data, and the equilibrium dissociation constants (Kd) were calculated from the obtained binding curves. As depicted in Figure 6, all bacteria exhibited nanomolar affinity for IL6, whereby the ethanol-killed cells had the lowest Kd of 3.23 nM, while the highest Kd of 4.89 nM was determined for the cells killed with heat at 100 °C. For other treated bacteria, calculated Kd values were in the range of 3.4-3.58 nM, comparable to that of non-treated live cells (Kd = 3.84 nM). Given that the interaction between IL6 receptor α and human IL6 has Kd of 9 nM (Boulanger et al., 2003), the estimated affinity suggest that the developed bacteria will be able to inhibit interaction between the IL6 and its receptor.

### 3.7 Ethanol-killed ZIL6-displaying *L. lactis* exhibits high maximum binding capacity for IL6 of 200 ng IL6 per mg dry cell weight, equal to that of live cells, while heat-killed bacteria retained 50% of maximum binding capacity of live cells

**Figure 7:**
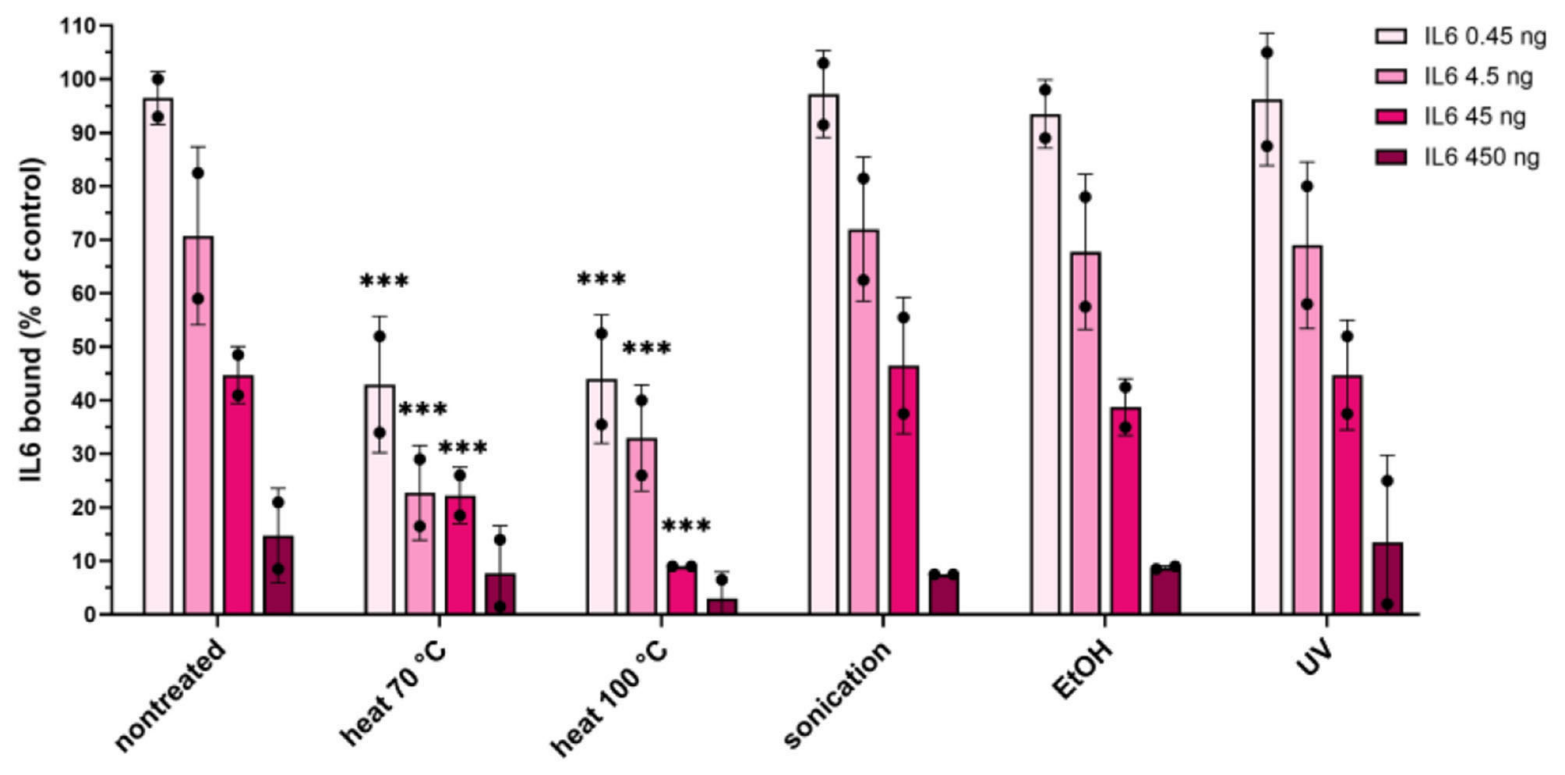
Quantification of the maximum binding capacity of ZIL6-displaying *L. lactis*. Increasing concentrations of recombinant human IL6 (0.45, 4.5, 45 and 450 ng) were incubated with a constant number of bacteria (4.5 × 10^7^ CFU equivalent to 0.1 mg dry cell weight) in 450 μl. Residual IL6 in the supernatants was quantified by ELISA for live non-treated bacteria and the cells treated by heat (70 °C, 40 min or 100 °C, 30 min), sonication, ethanol, and UV irradiation. The percentage of bound IL6 was calculated from the difference measured in the presence of ZIL6-displaying *L. lactis* and control bacteria (carrying empty plasmid pNZ8148). The data are means ± SD of two biological replicates. The asterisks denote statistically significant differences between treated and non-treated bacteria for each concentration. ***, *p* < 0.001 (one-way ANOVA with Dunnett multiple comparison test).

The maximum binding capacity of ZIL6-displaying *L. lactis* bacteria was quantified with ELISA as specified in Materials and Methods and the amount of bound IL6 per mg dry cell weight of bacteria was calculated. The dry cell weight (DCW) was inferred from optical density based on correlation: 1 OD_600_ = 0.3 g_DCW_/L, determined for *L. lactis* subsp. *cremoris* NZ3900 (Mierau et al., 2005). The results showed that 4.5 × 10^7^ CFU of live ZIL6-displaying *L. lactis* (equivalent to 0.1 mg dry cell weight) could remove 97% of IL6 from 0.45 ng amount in the solution, 70% of IL6 from 4.5 ng amount, and 45% of IL6 from 45 ng (Figure 7). Based on these results, the estimated maximum binding capacity of live ZIL6-displaying *L. lactis* is 200 ng IL6 per mg dry cell weight, considering the binding was proportional to bacterial concentration as demonstrated in our previous study (Zahirovic and Berlec, 2022). UV-treated, sonicated, and ethanol-killed cells were equally efficient as live non-treated bacteria in binding IL6, whereas the maximum binding capacity of heat-killed cells was 50% lower. Notably, the cells exposed to heat at 70 °C had a lower maximum binding capacity than non-treated bacteria, even though the affibody surface display on these cells was similar. This means that binders have remained on the cell surface after the treatment with heat at 70 °C, but a certain percentage of them may have been denatured and thus rendered non-functional. Therefore, among non-viable bacteria, ethanol-killed cells proved superior, showing comparable maximum binding capacity as live, non-treated cells. It is important to note that we used washed bacterial cells for the analysis, without spent growth medium. Hence, only the activity of the binders that were left on the bacterial surface after the treatment was determined. This excludes cellular components that may have been released by the treatments or during bacterial growth, which may enhance cytokine binding activity of treated cells or provide additional effects.

### 3.8 ZIL6-displaying *L. lactis* suppresses IL6-induced STAT3 signaling in HEKblue-IL6R cells in a dose-dependent manner, with up to 98% inhibition achieved by live bacteria and up to 78% by non-viable cells

**Figure 8:**
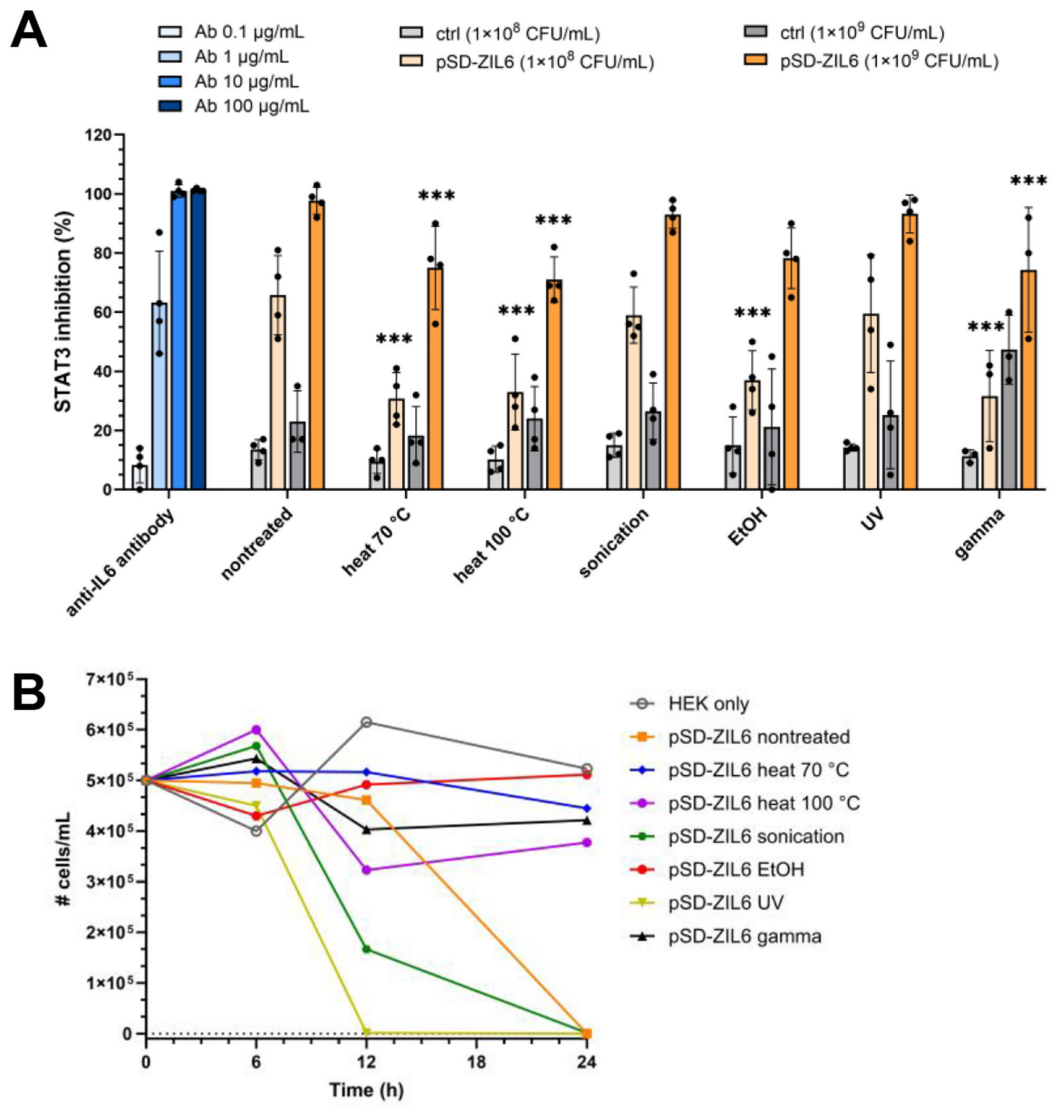
**(A)** Inhibition of STAT3 signaling in HEKblue-IL6R cells by non-viable ZIL6-displaying *L. lactis* bacteria in comparison to live non-treated strain. ZIL6-displaying *L. lactis* cells (1 × 10^8^ CFU/mL or 1 × 10^9^ CFU/mL) were preincubated with IL6 (1 ng/mL) for 2 h. After removal of bacterial cells, the cell-free supernatant containing remainder of IL6 was added to HEKblue-IL6R cells to induce the reporter system. The percentage of STAT3 inhibition was calculated relative to the STAT3 signaling induced by IL6 in the absence of bacteria. The inhibition of STAT3 signaling was determined for live non-treated bacteria and the cells treated by heat (70 °C, 40 min or 100 °C, 30 min), sonication, ethanol, UV, and gamma irradiation. pSD-ZIL6, *L. lactis* displaying ZIL6 affibody; Ctrl: *L. lactis* control cells containing empty plasmid pNZ8148. An anti-IL6 monoclonal antibody (Ab) was used as a positive control. The data are means ± SD of four biological replicates. The asterisks denote statistically significant differences between treated and non-treated bacteria for each concentration. ***, *p* < 0.001 (one-way ANOVA with Dunnett multiple comparison test). **(B)** The effect of lactic acid production by *L. lactis* on viability of HEKblue-IL6R cells. HEKblue-IL6R cells (100 000 cells/well) were incubated with ZIL6-displaying *L. lactis* (2 × 10^7^ bacteria/well) for 6, 12, and 24 h, and the viability of HEKblue-IL6R cells was determined with trypan blue. The data are means ± SD of three technical replicates of a representative experiment.

The biological activity of ZIL6-displaying *L. lactis* was determined by assessing the inhibition of IL6-mediated JAK/STAT3 signaling in HEKblue-IL6R cells. Stimulation of HEKblue-IL6R cells with increasing concentrations of IL6 showed a dose-dependent response (Supplementary Figure 6). For the inhibition experiment, previous studies have reported that long-term co-cultures of mammalian cells and live *L. lactis* (24 h and more) are not feasible due to medium acidification by live bacteria, which can negatively impact cell viability and thereby confound the results of host-microbe interaction assays (Taddese et al., 2021). Therefore, the inhibitory effect of bacteria on STAT3 signaling was examined by preincubation of IL6 with bacteria and subsequent stimulation of HEKblue-IL6R cells with the remaining IL6 in the cell-free supernatants. The preincubation with ZIL6-displaying *L. lactis* resulted in a dose-dependent suppression of STAT3 signaling in HEKblue-IL6R cells (Figure 8A). Specifically, at a concentration of 1 × 10^8^ CFU/mL, live non-treated bacteria suppressed 66% of STAT3 signaling, whereas heat-, ethanol-, and gamma-treated cells inhibited around 33% of STAT3 signaling. Inhibition achieved by sonicated and UV-treated cells was 59%, approaching that of non-treated bacteria. At a concentration of 1 × 10^9^ CFU/mL, the suppression was greater with both live and non-viable bacteria, whereby 98% inhibition of STAT3 signaling was achieved with non-treated, sonicated, and UV-irradiated cells and up to 78% with heat-, ethanol-, and gamma-treated cells. The inhibition efficiency of non-treated bacteria at 1 × 10^8^ and 1 × 10^9^ CFU/mL was equivalent to that of the anti-IL6 monoclonal antibody at 1 and 10 μg/mL, respectively. It is important to note that the treatment with higher concentration of empty plasmid control *L. lactis* cells also resulted in a certain percentage of inhibition of STAT3 signaling (around 45% for gamma-treated control cells and around 25% for other treated control bacteria), which can indicate non-specific effect on HEKblue-IL6R cells. This may be due to the acidification of cell culture medium by lactate from bacterial supernatants, which may have affected cell viability and thereby indirectly reduced SEAP secretion. We examined the influence of *L. lactis* on the viability of HEKblue-IL6R cells during 24 h co-culture. The assay showed that heat-, ethanol- and gamma-treated cells did not compromise the viability of HEKblue-IL6R cells, whereas non-treated, sonicated, and UV-irradiated bacteria led to the complete loss of cellular viability during 12–24 h of co-culture (Figure 8B). This confirms previous findings that lactate production and acidification of culture medium by metabolically active *L. lactis* cause mammalian cell death. Interestingly, gamma-killed bacteria did not influence the viability of HEKblue-IL6R cells, indicating that they have lost the ability to produce lactate, despite retaining membrane integrity and resazurin reducing ability. Besides control bacteria, pH-adjusted supernatants would be useful as additional control to provide information on the extent of the acidification effect.

In summary, our results showed that ZIL6-displaying *L. lactis* bacteria maintained their functionality and structure after the loss of microbial viability. The viability and the degree of functionality was dependent on type of the treatment that was used to kill bacteria. The effects of different treatments on viability, morphology and functionality of the analyzed bacteria are summarized in Table 2. Among non-viable cells, the ethanol-killed bacteria preserved the highest degree of functionality and displayed the same IL6 binding capacity as live strain, whereas heat-killed cells were somewhat less efficient and generally maintained 50% of the activity of live cells. The effect of 70 °C and 100 °C heat was examined to determine whether bacteria can preserve more functionality when exposed to lower temperature of 70 °C, that was still completely efficient at killing *L. lactis*. A lower temperature of 70 °C was not advantageous over 100 °C heat, since it did not result in retention of more activity in these bacteria. Given that the treatments were performed without a cytoprotective agent, it is conceivable to expect that the addition of protectants (e.g., trehalose) may result in greater functionality of heat-killed cells. Sonicated and UV-irradiated bacteria were equally effective as non-treated cells. However, sonicated bacteria were mostly alive; therefore unsuitable for preparation of postbiotic and also did not show any advantages over live non-treated bacteria in terms of functionality. On the other hand, the majority of the observed activity of UV-irradiated cells can be attributed to non-viable bacteria, since less than 10% of UV-irradiated cells were alive; therefore, UV-irradiated bacteria have great postbiotic potential provided that all cells are killed (e.g., by using higher UV intensity). Non-replicative gamma-irradiated bacteria retained ZIL6 affibody on the surface and exhibited inhibitory effect on IL6-mediated STAT3 signaling comparable to other non-viable cells, thereby demonstrating the suitability for further characterization. However, it is important to note that some metabolic enzymes appear to be active in gamma- and UV-irradiated cells, which warrants further investigation of their safety.

**Table 2:** A summary of the effects of the applied bacteria-killing treatments on recombinant *L. lactis* displaying ZIL6 affibody on their surface.

**Characteristic**	**Non-treated**	**Heat 70 °C**	**Heat 100 °C**	**Sonication**	**Ethanol**	**UV**	**Gamma**
Growth on agar plate	+	–	–	+	–	–/+	–
Cell membrane integrity	Intact	Damaged	Damaged	Intact	Damaged	Intact	Intact
Metabolic activity	+	–	–	+	–	+	+
Surface structure	Smooth	Dents	wrinkled	Deflated	wrinkled	Rough	Unaltered
Surface display of ZIL6 affibody	MFI = 345	MFI = 374	MFI = 205	MFI = 441	MFI = 827	MFI = 316	MFI = 305
Binding affinity (Kd)	3.84 nM	3.58 nM	4.89 nM	3.40 nM	3.23 nM	3.49 nM	n.d.
Maximum binding capacity	200 ng IL6/mg DCW	100 ng IL6/mg DCW	100 ng IL6/mg DCW	200 ng IL6/mg DCW	200 ng IL6/mg DCW	200 ng IL6/mg DCW	n.d.
STAT3 signaling inhibition	98%	78%	71%	93%	78%	93%	74%
Effect on HEK cell viability	+	–	–	+	–	+	–

+, presence of activity; –, lack of activity; n.d., not determined; MFI, mean fluorescence intensity; DCW, dry cell weight.

## 4 Discussion

Live probiotics have a long history of safe use in healthy population. However, administration of live bacteria to patients with a weak immune system or compromised intestinal barrier can have detrimental consequences, including infections and sepsis. Provided that functionality is retained, the use of postbiotic non-viable bacteria would represent a safer alternative. Non-viable forms of probiotic bacteria may retain similar activity as live strains, on the condition that their mechanism of action does not rely on microbial viability. Given that there are multiple potential mechanisms by which probiotics exert their action, the functionality of non-viable bacteria has to be demonstrated for each strain individually. When it comes to genetically engineered LAB, the question of whether non-viable bacteria can be utilized in therapy is rarely explored. For the first time, we investigated the functionality of non-viable recombinant *L. lactis* strains that are engineered to display binders of proinflammatory cytokines on their surface and are intended for the treatment of inflammatory intestinal diseases. We intentionally killed the bacteria for the purpose of improving safety and performed their structural and functional characterization in direct comparison to live cells. IL6 targeting *L. lactis* was engineered in our previous study by displaying ZIL6 affibody on the cell surface via non-covalent cell wall anchoring. The live bacteria showed high IL6-binding ability by removing clinically relevant amounts of IL6 *in vitro* (Zahirovic and Berlec, 2022). Here, we demonstrated that the cellular integrity, affibody surface display, binding affinity, maximum binding capacity, and biological activity are largely retained in ZIL6-displaying *L. lactis* after the bacteria have been killed by the applied treatments. The degree to which each function is preserved depends on the type of treatment, whereby ethanol-killed bacteria exhibited the greatest activity among non-viable cells.

Five different methods commonly used for killing bacteria were tested including heat, ethanol, sonication, UV or gamma irradiation to determine their effect on viability, morphology and functionality of analyzed bacterial strains and find out which method is most suitable for generation of postbiotics from recombinant *L. lactis*. Analysis of growth showed that bacteria were completely killed (unable to grow on agar plates) after exposure to heat, ethanol, and gamma irradiation. UV treatment, on the other hand, was partially effective as 6–8% of bacteria survived under the applied conditions. Complete killing by UV radiation can be achieved at a shorter irradiation distance (20 cm from the lamp), which provides higher UV intensity or by using a lower bacteria number. Sonication appeared least efficient and led to an increased number of colonies on agar plates as a result of disintegration of bacterial strings into individual cells. Given that the sonication induces bacterial death by causing mechanical and oxidative damage to the cell wall (Pandur et al., 2022), the higher resistance of *L. lactis* to sonication can be attributed to the thick layer of cross-linked peptidoglycan surrounding the surface of gram-positive bacteria, which provides protection against ultrasonic cavitation. Sonication is therefore not suitable for the generation of non-viable *L. lactis*.

The method used for the preparation of non-viable bacteria from recombinant strains may affect the structure and functionality of both bacterial cells and the expressed therapeutic moieties. In general, the functionality of bacteria that display binding proteins on their surface depends on (i) binder characteristics (affinity, specificity, stability), (ii) the extent of surface display of the binder (the amount of protein on the cell surface, accessibility of its binding sites for interaction with the target) and (iii) the characteristics of host bacterial strain (cell wall composition and structure, the presence of shielding molecules on the cell surface). These features were affected to varying degrees by the treatments applied to kill the bacteria in this study.

We initially included four recombinant *L. lactis* strains, each displaying a binding protein with a distinct scaffold (affibody, fynomer, or evasin) to determine whether the effect of the treatments is specific to each type of binder or uniform across different binders. The latter would indicate that the anchored proteins are protected by the confinement of the *L. lactis* cell wall. The analysis of the cytokine removal ability of bacteria showed that the treatments have varying impact on different binders. We observed poor resistance of evasin to most treatments, whilst fynomer was adversely impacted by gamma irradiation. In contrast, affibody was least affected, and both anti-IL6 and anti-TNF affibody-displaying *L. lactis* retained almost all of the cytokine binding ability of the live strain (at a cytokine concentration of 300 pg/mL). Based on these results, the IL6-targeting strain displaying ZIL6 affibody on the surface was selected for detailed characterization. Given that the influence of the treatments on the cytokine binding ability was similar in both affibody-displaying strains, it is conceivable to expect that our findings can be extended to other affibody-displaying bacteria. Regarding the protective effect of *L. lactis* cell wall on anchored proteins, previous studies have shown that some surface-displayed proteins are protected from harsh conditions in the environment, e.g., subtilisin QK-2 was more stable in simulated gastric fluid when anchored to the cell wall of *L. lactis*-derived bacterium-like particles than in a soluble form (Mao et al., 2016). In contrast, other studies have reported that cell anchoring alone does not provide sufficient protection against proteolytic denaturation, e.g., superoxide dismutase anchored to the cell wall of *L. fermentum* was digested by pepsin (Tay et al., 2020). For the protein binders employed in this study, no general protective effect of the cell wall was noticed; rather, the observed resistance of affibody-displaying strains could be ascribed to the inherent stability of affibody scaffold. Affibodies and fynomers are known as stable proteins (Skrlec et al., 2015). Affibodies, in particular, were found to be highly resistant to high temperatures (up to 90 °C), extreme acidic and alkaline conditions (pH from 2.5 to 11), and lipophilic organic solvents (Skrlec et al., 2015). This is attributed to their exceptional ability to refold with high fidelity within 3 μs after thermal or chemical denaturation (Arora et al., 2004). While some evasins were reported to be thermostable (e.g. evasin-4 melting temperature was ~80 °C) (Aryal et al., 2022), there is no data on stability of evasin-3. Our results indicate that evasin-3 has lower resistance to the applied treatments compared to affibody or fynomer. Binder stability aligned with secondary structure of their scaffold. Affibody scaffold is composed primarily of α-helices, while fynomers and evasins are made mostly of β-strands. Although there is no direct correlation between stability and percentage of β-strand and α-helix secondary structure in proteins, β-sheets are usually less stable than α-helices. This may be due to the fact the local interactions dominate in α-helices, as opposed to the long range contacts in β-sheets.

Regarding the effect of treatments on bacterial morphology, we observed small alterations in bacterial size and surface structure, which included cell shrinkage, dents, and wrinkling of the bacterial surface, while cell integrity was retained in all treated cells. Preserved cellular integrity may result in better activity of effector molecules due to increased avidity or prolonged residence time, as reported for vaccines (Kawana et al., 2014). The effect of the applied treatments on the integrity of cell membranes and the metabolic activity of *L. lactis* was consistent with their killing mechanisms. In fact, the major mechanism involved in the thermal death of bacteria is damage to the cell membrane and denaturation of proteins (Mitsuzawa et al., 2006). Similarly, ethanol kills bacteria by denaturing proteins and dissolving lipids within the cell wall and cell membrane (Kniggendorf et al., 2011). Unlike ethanol and heat, which act mostly on the bacterial envelope, UV and gamma irradiation kill bacteria by damaging DNA either directly or through the generation of reactive oxygen species (Porfiri et al., 2022). Accordingly, we found that the cell membranes of heat- and ethanol-killed *L. lactis* were damaged and the cells lacked metabolic activity. Conversely, UV- and gamma-irradiated *L. lactis* had intact membranes and a preserved ability to reduce resazurin. This is in line with other studies, which have found that LAB maintained residual metabolic activity after gamma or UV irradiation (van Tatenhove-Pel et al., 2019; Porfiri et al., 2022).

The changes in the morphology and physiology of bacterial cells, induced by various stressors (e.g., elevated temperature, low pH, high salts), can alter the configuration of surface-displayed proteins and thus impact their accessibility. For instance, exposure of *S. aureus* to NaCl (even at a low concentration of 1%) was found to decrease the number of antibody binding sites at the cell surface through ionic effects (Kennedy et al., 2019). Also, it has been reported that antibiotics (Zadravec et al., 2015), ethanol, and heat treatment (Taddese et al., 2021) can impact native surface proteins on *L. lactis*. When it comes to the surface display of ZIL6 affibody, we found that it was around 50% lower on heat-killed *L. lactis* (treated at 100 °C) compared to that on non-treated cells. This can be ascribed to the detachment of affibodies from the cell surface during heat exposure. By contrast, surface display of ZIL6 affibody was increased twofold in ethanol-killed cells, likely due to the removal of hindering molecules, such as lipoteichoic acids or other cell wall-tethered glycopolymers, from the bacterial surface by ethanol. It has been previously demonstrated that lipoteichoic acids shield LysM anchoring sites on the *L. lactis* surface (Steen et al., 2003). Additionally, a polysaccharide pellicle, which is present on the outer surface of *L. lactis*, may act as a barrier that prevents antibodies from reaching affibody epitopes in the cell wall (Chapot-Chartier et al., 2010). The pellicle might have been loosened or removed by the ethanol, resulting in an increased affibody exposure. Another commonly used method for preparation of postbiotics is crosslinking with formaldehyde, which can be tested in future studies to assess its influence on surface display and functionality of affibodies on *L. lactis* surface.

The extent of affibody surface display correlated with the binding affinity between bacteria and IL6. The enhanced ZIL6 affibody exposure on ethanol-killed bacteria led to an increased affinity of these cells for IL6 (Kd = 3.23 nM), whereas lower affibody surface display on the cells killed with heat at 100 °C resulted in lower binding affinity (Kd = 4.89 nM). In general, the differences in Kd values were relatively small, and all bacteria had strong (nanomolar) affinity for IL6. Furthermore, ZIL6-displaying *L. lactis* bacteria exhibited a high binding capacity of 200 ng IL6 per mg dry cell weight. The maximum binding capacity of UV-treated, sonicated, and ethanol-killed cells was comparable to that of live non-treated bacteria, whereas it was reduced by 50% in heat-killed cells. When compared to other IL6 adsorbents, this binding capacity is ~1,000 times greater than that reported for unfunctionalized polystyrene-CaCO_3_ adsorbent resin (Chai et al., 2019) or polymeric nanoparticles (Thamphiwatana et al., 2017). It is more than sufficient to allow the removal of excessive amounts of IL6 that are found in the intestine of IBD patients. Although the exact concentrations are not known, studies have reported elevated local levels of IL6, particularly in patients with active IBD. Early works reported the average amount of 54 pg IL6/mg protein in the intestinal mucosa of IBD patients, in contrast to 5 pg/mg protein in healthy individuals (Reimund et al., 1996). Also, the concentrations of up to 5,900 pg/mL IL6 have been measured in the cultures of mononuclear cells harvested from the inflamed tissues of IBD patients, in comparison to 57 pg/mL IL6 in control cells (Reinecker et al., 1993).

The removal of IL6 by bacteria led to the inhibition of STAT3 signaling in HEKblue-IL6R cells, indicating that ZIL6-displaying *L. lactis* might be an effective antagonist of IL6-induced signaling pathway. At a concentration of 1 × 10^8^ CFU/mL, preincubation of IL6 with non-viable bacteria resulted in 33% inhibition of IL6-induced STAT3 signaling, whilst 66% inhibition was achieved with live cells. When a tenfold higher concentration of bacteria was used, 78% inhibition of STAT3 signaling was observed with non-viable bacteria and 98% inhibition with live cells. Non-viable cells generally appeared less efficient in STAT3 signaling inhibition compared to live bacteria, probably due to the impact of medium acidification by live bacteria on HEKblue-IL6R cell viability. We observed that live and metabolically active bacteria (non-treated, sonicated, and UV-treated cells) led to the complete loss of cellular viability during 12–24 h of co-culture. Accordingly, the reduction of cell viability caused by lactate from the supernatants of live bacteria (used for stimulation of HEKblue-IL6R cells) may non-specifically reduce the amount of secreted SEAP due to a lower number of live HEKblue-IL6R cells in the culture. In addition, acidification of cell culture medium may reduce the activity of SEAP, which is optimal in an alkaline pH. The negative effect of lactate on mammalian cell viability can be circumvented by the use of non-viable bacteria devoid of metabolic activity. Hence, apart from safety advantages, non-viable bacteria can find practical use in co-culture assays with mammalian cells when long incubations are needed.

Overall, our findings demonstrated that functional non-viable bacteria can be obtained from ZIL6-displaying *L. lactis*. The results highlighted that the type of killing treatment plays an important role in shaping the functionality of obtained postbiotics. Although non-viable recombinant bacteria promise better safety profile, the chance of immune activation by the recombinant proteins on non-viable LAB surfaces remains, and will require additional evaluation. While *in vitro* results are promising, we acknowledge that *in vivo* validation is necessary to substantiate the findings and validate therapeutic potential of ZIL6-displaying postbiotics in inflammatory intestinal diseases.

## 5 Conclusion

In this study, we prepared postbiotics from IL6 targeting *L. lactis* displaying ZIL6 affibody on their surface with the long term aim to evaluate their therapeutic potential in inflammatory intestinal diseases and colorectal cancer. The results showed that surface-displayed ZIL6 affibody, non-covalently anchored to the cell wall of *L. lactis*, withstood the treatments applied to kill bacteria and remained functional after the loss of microbial viability. Ethanol-killed cells exhibited the greatest activity among non-viable bacteria. They exhibited the same high IL6-binding capacity as live strain (200 ng IL6/mg DCW), possessed strong (nanomolar) affinity for IL6, and were able to inhibit up to 78% of IL6-induced STAT3 signaling while showing enhanced affibody surface display. Hence, we obtained non-viable bacterial cells as delivery vehicles of IL6 targeting affibody into the GIT, which hold promise as a safe, stable, and effective alternative to live recombinant bacteria.

## Data Availability

The original contributions presented in the study are included in the article/Supplementary material, further inquiries can be directed to the corresponding author.
