# Supplementary material for: Postbiotics derived from recombinant lactic acid bacteria exhibit high IL6-binding capacity and suppress IL6-induced STAT3 signaling
Source: Front Microbiol. 2025 Oct 24;16:1657810. doi: 10.3389/fmicb.2025.1657810 (PMC12593480; doi:10.3389/fmicb.2025.1657810)
Supplement: Supplementary file 1 [file Supplementary_file_1.docx]

**Supplementary material**

**Postbiotics derived from recombinant lactic acid bacteria exhibit high IL6-binding capacity and suppress IL6-induced STAT3 signalling**

Abida Zahirović^1*^, Špela Zupančič^2^, Andraž Verdir^3^, Sebastjan Nemec^4#^, Slavko Kralj^2,4^, Luka Snoj^5^, Aleš Berlec^1,2^

*^1^Department of Biotechnology, Jožef Stefan Institute, Ljubljana, Slovenia*

*^2^Faculty of Pharmacy, University of Ljubljana, Ljubljana, Slovenia*

*^3^Reactor Infrastructure centre, Jožef Stefan Institute, Ljubljana, Slovenia*

*^4^Department for Material Synthesis, Jožef Stefan Institute, Ljubljana, Slovenia*

*^5^Reactor Physics Department, Jožef Stefan Institute, Ljubljana, Slovenia*

*Corresponding Author: [abida.zahirovic@ijs.si](mailto:abida.zahirovic@ijs.si)

#present address: Sebastjan Nemec, Materials Research Laboratory, University of Nova Gorica, Ajdovščina, Slovenia

**Suppl. Fig. 1 Nonviable bacteria did not recover their viability during a 14-day storage period.** The growth of ZIL6-displaying *L. lactis* on solid and liquid medium determined first day upon treatments and after 14 days of storage. pSD-ZIL6, *L. lactis* displaying anti-IL6 affibody; pNZ8148, *L. lactis* harbouring empty plasmid.

**Suppl. Fig. 2  Single-staining of ZIL6-displaying *L. lactis* with SYTO 9.** Comparison of the intensity of SYTO 9 fluorescence between treated and nontreated cells.

**Suppl. Fig. 3 Violin plots showing the cell size distribution of ZIL6-displaying *L. lactis* bacteria before and after the treatments.** The size of 50 individual bacterial cells on SEM images was measured for each treatment using ImageJ 1.52g. Dashed lines within the violins indicate the 1st quartile, median, and 3rd quartile values.

**Suppl. Fig. 4 Extracellular DNA (eDNA) release.** DNA concentrations were measured by Nanodrop (at 260 nm) and presented as mean ± SD of three technical replicates across all samples. **, p = 0.007; ***, p < 0.001 (one-way ANOVA with Dunnett multiple comparison test).

**Suppl. Fig. 5 Time course of IL6 binding to ZIL6-displaying *L. lactis***. Bacterial suspension containing 1×10^8^ CFU/mL of ZIL6-displaying *L. lactis* was incubated with recombinant human IL6 (1000 pg/mL) for indicated time intervals. Following incubation, bacterial cells were removed by centrifugation and the remainder of IL6 was quantified for each time point by ELISA. The percentage of bound IL6 was calculated and plotted as a function of time. Data are means ± standard deviation of two technical replicates of a representative experiment. pSD-ZILf, *L. lactis* harbouring plasmid for surface display of anti-IL6 affibody; ctrl: *L. lactis* control cells containing empty plasmid pNZ8148.

**Suppl. Fig. 6 Dose-response of HEK-Blue IL-6 cells to recombinant human IL6.** Cells were stimulated with increasing concentrations of recombinant human IL6. After overnight incubation, IL6-induced STAT3 signalling was determined by measuring the amount of secreted embryonic alkaline phosphatase (SEAP) in the cell culture supernatant using QUANTIBlue Solution.
